# The College of Nursing, Limited: Constitution of the Scottish Board

**Published:** 1916-10-14

**Authors:** 


### THE MATRONS' AND SISTERS' DEPARTMENT.

### THE COLLEGE OF NURSING, LIMITED.

### Constitution of the Scottish Board.

THE Council of the College of Nursing at its meeting on September 21 approved the establishment of a Local Board for Scotland, to be designated the Scottish Board, for the superintendence of the affairs of the College of Nursing in Scotland, under Sections 79 to 82 of its Articles of Association. The provisions of the Scottish Board's constitution are as follows:—

THE FIRST BOARD.

That the first Scottish Board be composed of thirty members. The following have so far consented to serve:—

Miss Gill, lady superintendent of nurses, Royal Infirmary, Edinburgh; Miss Melrose, matron, Royal Infirmary, Glasgow; Professor Glaister, M.D., D.P.H. (Camb.), F.R.S.Edin., University of Glasgow; Miss Pegg, matron, Royal Infirmary, Dundee; Miss Merchant, matron, Eastern District Hospital, Glasgow; Colonel Roxburgh, convener House Committee, Western Infirmary, Glasgow; Dr. Maxtone Thom, D.P.H., superintendent, Royal Infirmary, Glasgow; Miss Graham, Scottish Nurses' Association, Edinburgh; Miss Campbell, matron, Victoria Infirmary, Glasgow; Dr. Claude Ker, general superintendent, City Fever Hospital, Edinburgh; Mr. C. C. Barrie, chairman, Royal Infirmary, Dundee; Miss Annie Peterkin, general superintendent, Queen Victoria's Jubilee Institute, Scottish Branch; Colonel Mackintosh, M.V.O., M.B., LL.D., superintendent, Western Infirmary, Glasgow; Sir James Affleck, M.D., consulting physician, Royal Infirmary, Edinburgh. Seven members to be co-opted shall be nurses. Casual vacancies shall be filled by co-optation.

SCOTLAND'S REPRESENTATION ON COLLEGE COUNCIL.

That the proportion of members of the Council of the College representing Scotland be one-sixth; that they shall be nominated by the Scottish section of the Register, and shall be elected by postal ballot, as laid down in Paragraph 41 of the Amended Articles of Association; that two shall retire in 1918, two in 1919, and two in 1920, and shall be eligible for re-election.

COMPOSITION OF SCOTTISH BOARDS AFTER 1918.

That unless the Council shall upon the recommendation of the first Scottish Board otherwise determine, the Scottish Board after the ordinary general meeting of the College in 1918 shall consist of thirty members, and shall be made up as follows:—

(*a*) One member shall be elected by each of the following bodies:—Royal College of Physicians, Edinburgh; Royal College of Surgeons, Edinburgh; Royal Faculty of Physicians and Surgeons, Glasgow.

(*b*) Six members shall be elected for periods of three years by the Boards of the following hospitals (one representative being elected by each Board), viz.: The Royal Infirmary, Edinburgh; the Royal Infirmary, Glasgow; the Royal Infirmary, Aberdeen; the Royal Infirmary, Dundee; the Victoria Infirmary, Glasgow; the Western Infirmary, Glasgow.

(*c*) The six Scottish members of the Council of the College shall sit on the Scottish Board *ex-officio*.

(*d*) Fifteen members shall be nominated and elected (by postal ballot when required) by the members of the College resident in Scotland. Of these, not fewer than two shall be superintendents, and not fewer than two matrons of Scottish hospitals. Of the members elected in 1918, five shall retire in 1919, five in 1920, and five in 1921, and thereafter in that proportion. From 1919 onwards the members elected each year shall hold office for three years, and shall be eligible for re-election.

Should any casual vacancy occur among the elected members the Board shall elect a member of the College to hold office for the remainder of the period of the said member.

REPRESENTATION ON STANDING COMMITTEES.

That every standing committee of the Council shall include at least one Scottish representative in six.

REGULATION OF PROCEEDINGS.

That the Scottish Board shall be free to regulate its meetings and proceedings subject to the approval of the Council.

REPORTS TO THE COUNCIL.

That the Scottish Board shall from time to time report to the Council through the Consultative Committee what hospitals in Scotland possess nursing schools whose training may be accepted as qualifying for the Register of the College.

That the Scottish Board shall report to the Council, through the College Registration Committee, as to the qualifications of applicants for membership of the College, who have already completed their training in Scotland at the date of the establishment of the College.

EXAMINATIONS.

That all examinations in Scottish nursing schools shall be conducted in conformity with the standard of examination determined by the College Council, by examiners appointed by the Council upon the nomination of the Scottish Board through the Examination Committee.

OFFICE IN SCOTLAND.

That the Scottish Board shall be provided with a local Secretary resident in Scotland, and with an office, the expenses to be defrayed by the College. The Secretary shall be appointed by the Council upon the nomination of the Scottish Board, and shall carry out such duties as the Board may arrange.

ANNUAL ESTIMATE OF EXPENSES.

That the Scottish Board shall annually prepare and send to the Secretary of the Finance Committee an estimate under various heads of the moneys required for expenditure by the Board, including travelling expenses of members to London.

**COLLEGE OF NURSING SCOTTISH BOARD**—(*Continued.*)

That the estimate, when approved by the Council, and the amounts included therein, shall be appropriated to the several objects specified in the estimate, and the Scottish Board shall be authorised to expend sums not exceeding such amounts during the financial year for the several objects so specified. No expenditure shall be incurred by the Board except such as is provided for in the annual vote. Any proposed expenditure not so provided for shall be the subject of a special estimate submitted by the Scottish Board to the Council through the Finance Committee.

That periodical returns of the expenditure incurred shall be submitted to the Finance Committee, and the Finance Committee shall present to the Council at the termination of each financial year a statement thereon with such comments as they may think advisable.

